# Three Patients With Chorea and Positive Voltage-Gated Potassium Channel Antibody: Is This the Link Between Hyperkinetic von Economo Disease and COVID-19?

**DOI:** 10.7759/cureus.35666

**Published:** 2023-03-01

**Authors:** Jacqueline Dulanto, David Chu, Pardis Saffari, Mina Abdelshahid, Prissilla Xu, Jacob Hauser, Jonathan Eskenazi, Lynnea Morm, Antonio K Liu

**Affiliations:** 1 Internal Medicine, Adventist Health White Memorial, Los Angeles, USA; 2 Family Medicine, AltaMed Health Services, Los Angeles, USA; 3 Pharmacy Services, Adventist Health White Memorial, Los Angeles, USA; 4 Neurology, Adventist Health White Memorial, Los Angeles, USA; 5 Neurology, California Hospital Medical Center, Los Angeles, USA; 6 Neurology, Loma Linda University School of Medicine, Loma Linda, USA

**Keywords:** covid-19, voltage-gated potassium channel antibody, postinfectious, chorea, von economo disease

## Abstract

Chorea, hemichorea, and other movement disorders have been reported after different pandemics since Constantin von Economo’s time. In the current COVID-19 pandemic, numerous delayed neurological manifestations have been reported in the postinfectious or postvaccination periods. However, very few of these are movement disorders in nature; there are even fewer voltage-gated potassium channel (VGKC) antibody-related movement disorder cases in the literature. We encountered three patients with some COVID-19-related issues featuring both chorea and VGKC antibody. Modern medical science and technology may be able to further our understanding of the molecular basis of von Economo disease and reveal a possible link to COVID-19 along with the immunomodulation aspect of its treatment.

## Introduction

In 1920, two years after the 1918 Spanish flu, Austrian neurologist Constantin von Economo reported patients with movement disorder that eventually came to be known as von Economo disease. von Economo disease, von Economo encephalitis, or encephalitis lethargica encompassed a variety of movement-related symptoms that are generally believed to be autoimmune and postinfectious in nature. Clinically, there are three forms: the somnolent-ophthalmologic, amyostatic-akinetic, and hyperkinetic forms [1]. Despite being temporally related to the 1918 Spanish flu, a conclusive causative agent has never been identified [2]. A literature search on the subject matter using the keywords “COVID-19” and “von Economo” yielded four papers reporting observational post-COVID-19 movement disorders [3-6]. There was little discussion on pathophysiology, molecular mimicry, or treatment.

Neurologists have compared and contrasted von Economo disease with postencephalitic parkinsonism before the current COVID-19 pandemic [7]. Additionally, there are a few observational case series that predict the rise of post-COVID-19 parkinsonism [8-10]. Few, however, mentioned “von Economo” in their papers, and there was no discussion on antibody testing.

Linking autoimmune antibodies to movement disorder is not novel. A large-scale study on 844 patients has found many autoimmune antibodies being positive for a variety of movement disorders, among them included amphiphysin, glutamic acid decarboxylase (GAD), N-methyl-D-aspartate receptor (NMDAR), and leucine-rich glioma inactivated 1 (LGI1) antibodies. Voltage-gated potassium channel (VGKC) was not a usual finding [11].

VGKC’s role in movement disorders is generally paraneoplastic in nature, such as in Morvan’s syndrome [12]. The movement disorder of Morvan’s syndrome is described as peripheral nerve excitability and myokymia, but specifically, there is no mention of chorea. Without malignancy, VGKC’s role in movement disorder is less well-established, to begin with [13]. VGKC has a wider recognition in causing encephalitis [14]. So far, VGKC’s possible association with chorea as a post-COVID-19 complication has not been reported.

We reported three patients who developed choreiform movement with positive VGKC antibodies in the past eight months. One patient had received a COVID-19 mRNA Moderna booster shot 14 days prior. Another patient tested positive for COVID-19 on nasal swab PCR three weeks prior to presentation. The last patient never tested positive for COVID-19 but was living among close family members who were COVID-19-positive. Besides the immunological profile, their treatment response was interesting and will be explored in the Discussion section. These cases suggest that there could be a molecular link between COVID-19 and postinfectious chorea (von Economo disease type of occurrence).

## Case presentation

Case 1

Patient 1 was a 57-year-old female with a past medical history of type 2 diabetes mellitus, hypertension, dyslipidemia, gastritis, major depressive disorder, generalized anxiety disorder, and chronic substance abuse (smoking methamphetamine). She received her second Moderna booster 14 days prior to presentation. Five days prior to presentation, she developed involuntary body movements. The movements were a combination of myoclonus at rest, dystonia when she tried to hold objects, and an otherwise continuous chorea movement. She also has akathisias (inability to stay motionless). It progressively worsened, and she became bedridden secondary to her inability to stand; the chorea had affected her lower extremities. She was also dysarthric, dysphagic, and unable to use her limbs functionally. Despite her motor decline, she remained oriented and coherent throughout; she was constantly involved in intelligent and meaningful discussions of her diagnosis and management. A trial of various medications led to the discovery that her symptoms were only responsive to the dopaminergic agent ropinirole. MRI of the brain with and without contrast showed no significant findings. Cerebral spinal fluid (CSF) analysis showed normal white blood cells but elevated protein. She tested positive for VGKC and GAD antibodies (Table 1). Her symptoms (both chorea and myoclonus) improved after methylprednisolone, plasmapheresis (once every other day for five cycles), and a course of rituximab 375 mg/m^2^. By the time she was discharged home, after 45 days, she was able to feed herself, use the bathroom with minimal assistance, and ambulate with a walker. She was discharged on low-dose ropinirole.

**Table 1:** Patient and laboratory characteristics of three cases of chorea with positive VGKC antibody. GAD: glutamic acid decarboxylase antibody, VGKC: voltage-gated potassium channel antibody, COVID: coronavirus disease, GD1b: ganglioside-disialic 1b antibody, WBC: white blood cell, MRI: magnetic resonance imaging

	Case 1	Case 2	Case 3
Age/sex	57/female	30/male	78/male
COVID-19 history	No known infection	Positive nasal swab one month prior	No known infection, but multiple family members tested positive within the last month
COVID-19 vaccination	Moderna second shot 14 days prior	Not vaccinated	Vaccinated twice, six months prior
Medical history	Diabetes, hypertension, methamphetamine use	Cocaine use	Diabetes, hypertension
Neurological presentation	No altered mental status, generalized chorea, myoclonus, dystonia, and akathisias	Worsening psychosis, minimal altered mental status, ataxia, chorea, myoclonus	Minimal altered mental status, left-sided chorea, sparing the right
Serum VGKC antibody (0-31)	104 pmol/L	59 pmol/L	41 pmol/L
Serum GAD antibody (0-5)	>250 IU	>250 IU	0
Other laboratory tests	Non-revealing	GD1b: 55	Non-revealing
Cerebral spinal fluid	Open pressure: 17 cm, WBC: 4/mm^3^ (0-8), protein: 193 mg/dL (15-45)	Open pressure: 23 cm, WBC: 0/mm^3^, protein: 38 mg/dL	Open pressure: not recorded, WBC: 6/mm^3^, protein: 40 mg/dL
MRI brain with and without contrast	Non-revealing	Non-revealing	T1 right basal ganglia lesion
Symptomatic treatment	Ropinirole	Baclofen	Olanzapine
Immunological treatment	Methylprednisolone, plasma exchange, rituximab (once)	Methylprednisolone, IV immunoglobulin, rituximab (once), cyclophosphamide	Methylprednisolone, IV immunoglobulin, rituximab (once)
Outcome	Chorea subsiding, ambulation with a walker at 45 days	Chorea subsided, ambulation with a cane at four months	Chorea subsiding, ambulation with a four-point cane at six weeks

Case 2

Patient 2 was a 30-year-old male with a history of recreational drug use (snorting methamphetamine) presenting with involuntary chorea and muscle spasms. He tested positive for COVID-19 nasal swab three weeks prior to presentation. On admission, he tested negative for COVID-19. On examination, the patient appeared to be coherent and oriented with mostly upper extremity generalized chorea. His initial motor strength was 5/5 in all four extremities. He had a stiff, spastic gait and dysdiadochokinesia. After trials of many medications, his chorea was found to be responsive only to baclofen. Unfortunately, his symptoms progressed, and he fell constantly when he stood up. MRI of the brain with and without contrast was unremarkable, and his CSF analysis showed 0 WBC and protein of 38 mg/dL. He was HIV-negative. Laboratory workups including B12 level and thyroid-stimulating hormone (TSH) were normal. The patient was started on intravenous immunoglobulin (IVIG) 400 mg/kg/day for five days and methylprednisolone, and symptoms improved. However, he experienced a relapse one month later, and laboratory results showed elevated titers: ganglioside-disialic 1b antibody (GD1b) of 55, GAD of >250 IU/mL, and VGKC of 59 pmol/L (Table 1). MRI of the brain with and without contrast at the time of his relapse was also negative. A second round of IVIG (same dose) was started in addition to one dose of rituximab 375 mg/m^2^ and cyclophosphamide. The patient’s symptoms finally improved again, and he was able to ambulate with a cane by the time of discharge four months after symptoms first started.

Case 3

**Figure 1:**
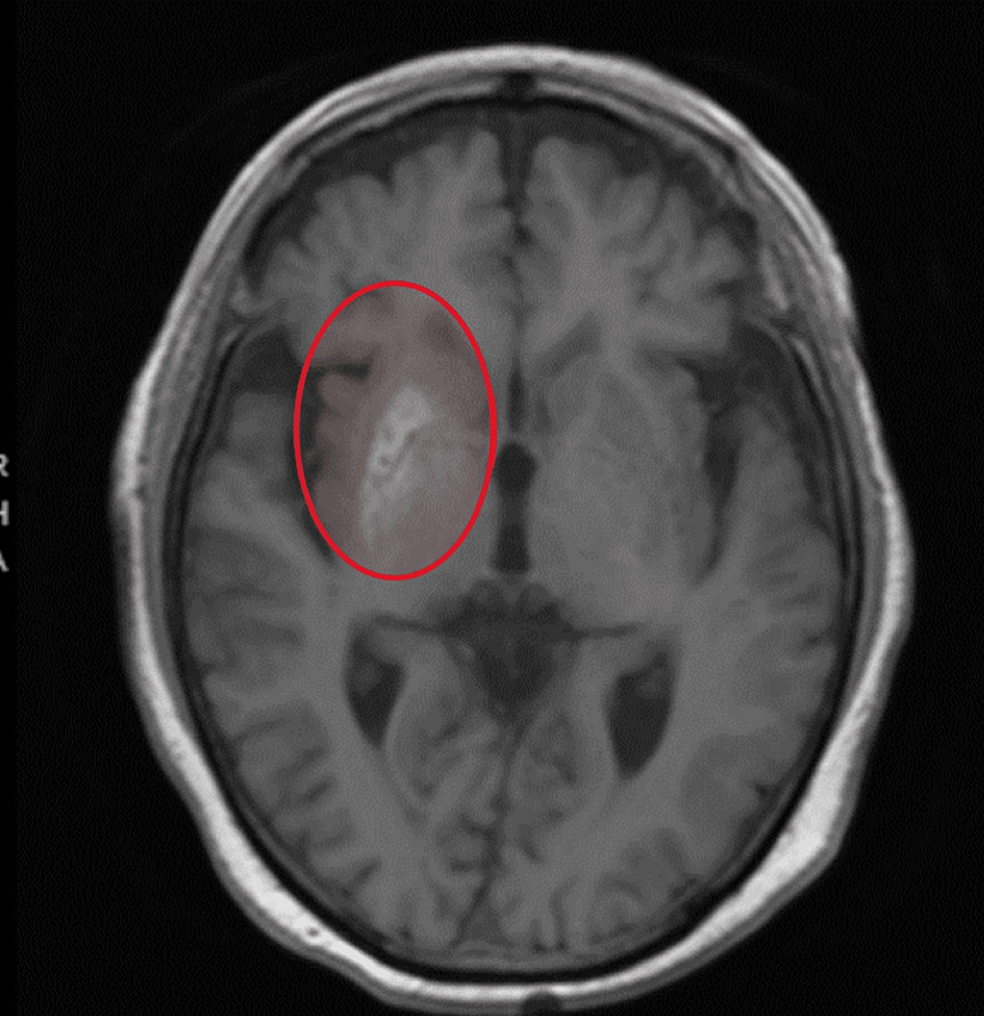
MRI T1 signal abnormality of the right basal ganglia (red oval). All other sequences were negative. MRI: magnetic resonance imaging

Patient 3 was a 78-year-old male with a history of type 2 diabetes mellitus and benign prostatic hypertrophy who presented to the hospital with one week of progressive involuntary movement of the left upper and lower extremities. He had a negative COVID-19 screening test, but he was living with several COVID-19-positive family members in the past month. He had never shown any signs of viral illness. The movement issues progressed to become frank chorea of the whole left side of the body. The right side was spared with no associated weakness, and he had normal mentation throughout. His symptoms progressed to the point that he could no longer even support himself standing, and he eventually lost any functional use of his left side. Laboratory workups including heavy metal screens were negative. MRI of the brain with and without contrast revealed no abnormal findings on diffusion-weighted imaging, fluid-attenuated inversion recovery (FLAIR), or T2 sequences. There was an abnormal signal on T1 at the right basal ganglia with no contrast enhancement (Figure 1). CSF analysis was negative. He was found to have an elevated VGKC antibody (Table 1). A trial of medications revealed that the chorea only responded to olanzapine at 20 mg daily. The patient was started on methylprednisolone, IVIG 400 mg/kg/day for five days, and one dose of rituximab 375 mg/m^2^. His symptoms began to improve 30 days after admission. Six weeks later, while he was at the nursing facility, he was able to ambulate independently with a four-point cane and hold objects briefly in his left hand. MRI of the brain repeated at that point shows no progression or resolution of the right basal ganglia lesion.

## Discussion

Overall, there are only a few articles reporting a movement disorder that developed after COVID-19 infection or COVID-19 vaccination. We have reported three patients with chorea with positive VGKC antibodies. The first had the Moderna COVID-19 mRNA booster shot, of which chorea had not been reported as a sequela. One was infected by COVID-19 and subsequently had a minor viral course. The last one, despite testing negative, had known exposure. We believe that these cases of chorea may be COVID-19-related. It is impossible to prove in a case report setting that a cause-and-effect relationship is conclusive. We are merely explaining a possible association for future observation and data gathering/analysis.

Besides a possible COVID-19 relationship, the three cases share further similarities with important distinctions. Firstly, they all bear a positive VGKC antibody. Cases 1 and 2 also tested positive for GAD antibodies. However, case 3 had no GAD antibody identified. GAD and VGKC are not commonly positive together, like in the case of limbic-like encephalitis [13]. GAD itself, more often than VGKC, has been more frequently associated with a movement disorder, from ataxia to stiff-person syndrome [14,15]. However, in our current report, VGKC is the common link. Some movement disorder experts have considered that VGKC lacks sensitivity and specificity [16]. Being a case report of only three patients, we can neither support nor disagree with these expert opinions; our purpose is to show that VGKC may have an unexplained role in the pathophysiology of viral-mediated movement disorder. Interestingly, GD1b is also positive in case 2. GD1b antibody has a known association with parkinsonism [17,18], but its affiliation with chorea or hyperkinetic state is not clear.

From a workup standpoint, all three patients had normal CSF white blood count. Two of the patients had normal CSF proteins. All other CSF tests including infection studies, myelin basic protein, and oligoclonal bands were all negative. Only one MRI of the brain showed abnormal signal density on T1 sequence; it is only on one side. There was no diffusion-weighted imaging, fluid-attenuated inversion recovery (FLAIR), or post-contrast in all three patients’ initial and follow-up MRIs. The T1 abnormality in case 3 sat at the right basal ganglia, making sense from a localization standpoint as a right basal ganglia lesion can certainly be responsible for a left body chorea or movement abnormality. However, T1 brightness usually suggests blood products or fat. Since CT was negative for blood and all the other MRI sequences remained negative, the clinical significance of this finding was nonspecific. T1 abnormalities in VGKC limbic encephalitis are usually associated with T2 and FLAIR abnormality at the temporal lobe and hippocampal area [19]. Therefore, our current MRI finding on case 3 from an autoimmune aspect is novel and will need further investigation.

For the autoimmune antibody detection, we used the autoimmune neurological disease reflexive panel of the Associated Regional and University Pathologists, Inc. (ARUP) Laboratories [20]. The test carries 16 antibodies commonly associated with neurological diseases. It is not a common first-round neurological workup employed by clinicians. It takes an average of 18-22 days to run, which means that it usually fulfills a confirmation role rather than providing “real-time” information to aid management. The challenge to managing these patients remains in arriving at the diagnosis with a high degree of suspicion and initiating treatment when the diagnosis remains only a differential.

Treatment for these three patients consisted of both symptomatic treatment and immunomodulation/immunosuppression. Symptomatic treatment was challenging in that it was primarily trial and error as there was no standard of care to follow. However, symptoms greatly improved upon starting one agent, suggesting some level of responsiveness. We confirmed the responsiveness by observing the recurrence of symptoms by halting treatment. Medications administered varied from sedative, dopaminergic, muscle relaxant, antiepileptic, and typical and atypical antipsychotics. Tetrabenazine and deutetrabenazine were not available as inpatient medications. All three patients responded differently. The patient who developed chorea after the Moderna mRNA vaccine booster responded mainly to the dopaminergic agent. This did not come as a surprise as dopamine-responsive chorea and movement disorder have been reported previously [21]. Dopamine agonists play a pivotal role in von Economo encephalitis with parkinsonism [22]. It is postulated that levodopa acts in a similar way as amantadine in enhancing dopaminergic transmission in the basal ganglia, thereby improving dystonia and other extrapyramidal movements [23]. The other two patients had no response to dopaminergic agents. The patient who had chorea with VGKC, GAD, and GD1b antibodies only responded to baclofen. Unlike dopaminergic agents, there are fewer reports of baclofen as a treatment in chorea. Baclofen, an analog of gamma-aminobutyric acid, is presumed to have helped as the compound is known to be deficient in Huntington’s chorea [24,25]. The last patient who had an abnormal lesion at the right basal ganglia responded symptomatically to olanzapine. Olanzapine has been commonly studied and tried on patients with chorea and is the most used antidopaminergic agent for chorea in the United Kingdom [26]. The patient’s response indicated that the underlying mechanism might be closer to Huntington’s chorea. These different responses suggest that the underlying pathophysiology may be different despite VGKC-positive antibodies and that chorea is just the predominant bedside presentation. All three patients responded symptomatically to some medication, but none of these medications had direct relationships with VGKC reported in the literature.

As there is a limited amount of therapeutics available, the primary challenge is determining whether or not our selected therapy has made an impact. We are not dealing with well-known autoimmune diseases (such as Guillain-Barré syndrome), where the time frame of progression, plateau, and recovery have been relatively well-studied [27]. We also do not know the time frame of our disease entity. It may be monophasic or polyphasic if early treatment and immunotherapy were not administered. All three patients were adults without evidence of streptococcal or other infections except COVID-19. All three patients received Solu-Medrol, intravenous immunoglobulin, rituximab, and commonly suggested therapies [28]. Ultimately, all patients improved about two weeks after the initiation of immunosuppressive therapy, and two out of the three patients regained functional status by the end of the third month. Movement disorders as a sequela of COVID-19 infection or mRNA COVID-19 vaccination are rarely reported. Three were seen within a short period of time at this community-based teaching hospital. A unique feature is that they were all VGKC antibody-positive, pointing toward autoimmunity and molecular mimicry as part of the pathophysiology. Two cases were GAD-positive, and one was also GD1b-positive. In Constantin von Economo’s time, modern imaging, CSF analysis, autoimmune antibody, and symptomatic or immunomodulation medications were not at his disposal. Recently, some practitioners have already observed and even predicted a rise in post-COVID-19 parkinsonism and movement disorder. From our observation, VGKC could play a role in molecular pathophysiology.

## Conclusions

The short-term and long-term effects of COVID-19 infection and mRNA vaccine in the human body are still unknown. It is only through rigorous reporting and analysis of data that a firm relationship between these movement disorders and COVID-19 can be elucidated epidemiologically. This reporting may link strange presentations with potentially treatable immunological processes. The current observations, as others have pointed out, may turn out to be von Economo disease of the 21st century. There is a possibility that this time around, equipped with current science and through a globally interconnected community, we can confirm COVID-19 as one of the pathogens involved in developing von Economo disease.
